# Syndrome of inappropriate antidiuresis/hyponatremia in COVID-19

**DOI:** 10.1007/s11102-024-01446-4

**Published:** 2024-08-28

**Authors:** Alessandro Peri, Laura Naldi, Dario Norello, Benedetta Fibbi

**Affiliations:** 1 Pituitary Diseases and Sodium Alterations Unit, AOU Careggi, Florence, 50139 Italy; 2 Endocrinology, Department of Experimental and Clinical Biomedical Sciences “Mario Serio”, University of Florence, AOU Careggi, Viale Pieraccini, 6, Florence, 50139 Italy

**Keywords:** Hyponatremia, Syndrome of inappropriate antidiuresis (SIAD), COVID-19, Outcome

## Abstract

Hyponatremia is the most frequent electrolyte alteration among hospitalized patients and it has been reported in 20–40% of patients with SARS-CoV-2 (COVID-19) infection. Multiple causes of hyponatremia have been hypothesized in these patients. The syndrome of inappropriate antidiuresis (SIAD) has been considered one of the main reasons leading to hyponatremia in this condition. SIAD can be secondary to cytokines release, in particular IL-6. Positive pressure ventilation can be another cause of hyponatremia due to SIAD. Other possible etiologies of hyponatremia in COVID-19 patients can be related to secondary hypocortisolism, nausea, vomiting, heart and kidney damage. Similar to many other clinical conditions, there is strong evidence that hyponatremia is associated with a worse prognosis also in patients with COVID-19 infection. In particular, hyponatremia has been identified as an independent risk of ICU transfer, need of non-invasive ventilation and death. Hyponatremia in COVID-19 patients is in principle acute and symptomatic and should be treated as such, according to the published guidelines. Therefore, patients should be initially treated with i.v. hypertonic saline (3% NaCl) infusion and serum [Na^+^] should be frequently monitored, in order to remain within a safe rate of correction. There is evidence showing that serum [Na^+^] correction is associated with a better outcome in different pathologies, including COVID-19 infection.

## Introduction

Hyponatremia is the most common electrolyte disorder among hospitalized patients. Based on the reduction of serum sodium concentration ([Na^+^]), mild hyponatremia (serum [Na^+^] between 130 and 135 mEq/L) occurs in about 20% of patients, and moderate/severe hyponatremia (serum [Na^+^] < 130 mEq/L) is found in up to 7% of patients [1–3]. Such a frequent occurrence is not surprising, if the different pathological conditions that can be associated with this electrolyte alteration are considered. Admittedly, hyponatremia encompasses a broad spectrum of different situations encountered in clinical practice. Essentially, hyponatremia covers every field of medicine.

**Fig. 1:**
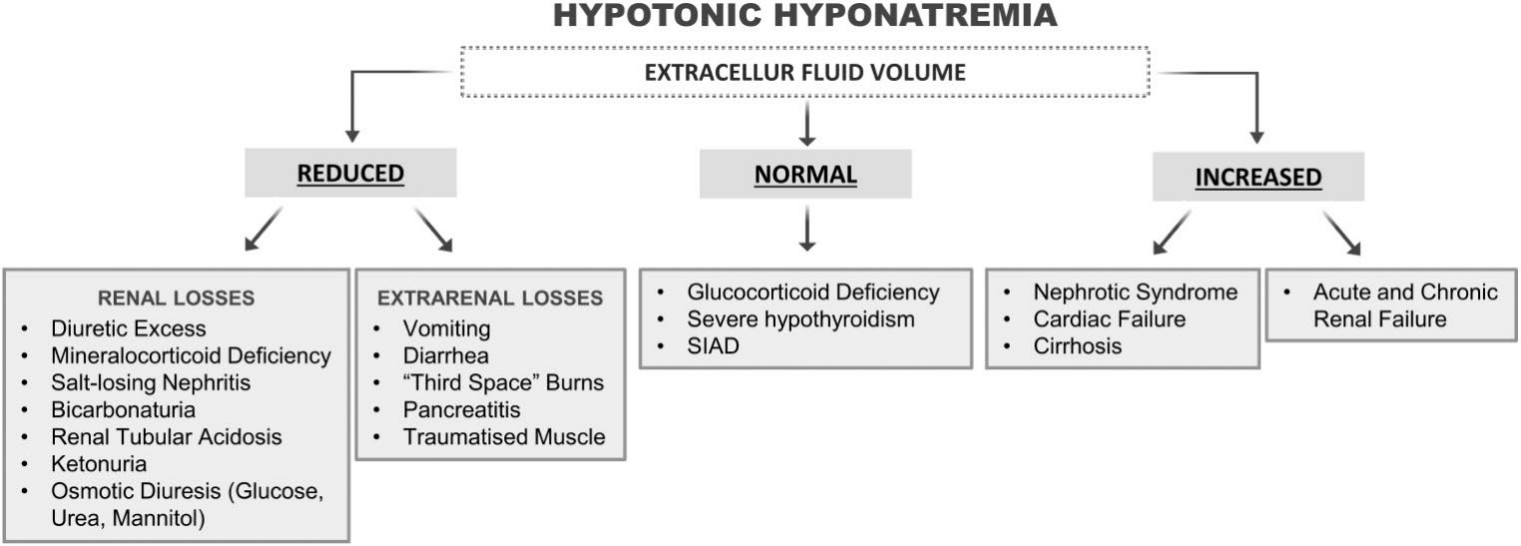
Etiology of hypotonic hyponatremia, according to the extracellular volume status

According to serum osmolality, hyponatremia can be divided into hypotonic and non-hypotonic forms [4]. Non-hypotonic hyponatremias include hypertonic hyponatremia, also defined as translational hyponatremia, isotonic hyponatremia, and pseudohyponatremia [4, 5]. Hypertonic hyponatremia is the result of a shift of water from cells into the extracellular fluid. In case of hyperglycemia, for instance, this shift is driven by the accumulation of glucose into the extracellular compartment. Isotonic hyponatremia may occur as a consequence of the retention of isotonic fluids that do not contain sodium into the extracellular space. This situation may be secondary to the infusion of large volumes of isotonic fluids during uro/gynecological surgery. Finally, pseudohyponatremia is the result of a laboratory artifact that occurs in the presence of severe hypertriglyceridemia or paraproteinemia, when serum [Na^+^] is measured by flame photometry [4, 5]. Hypotonic hyponatremia, also indicated as dilutional hyponatremia, is characterized by an excess of water in relation to sodium. This situation is most often due to impaired renal excretion of water and less frequently to an excess of water intake in a short period of time (i.e. water intoxication). Patients, in which hyponatremia is a consequence of impaired renal excretion of water, can be divided into three categories, according to the extracellular fluid volume (EFV), which can be reduced, normal or increased [6] (Fig. 1). To address EFV instead of volemia appears more appropriate to describe different forms of hyponatremia, because the former includes both the circulating volume and the interstitial fluids. Therefore, in this review the terms hyponatremia with reduced, normal or increased EFV will be used instead of the most commonly used hypovolemic, normovolemic and hypervolemic hyponatremia.

One of the most common forms of hyponatremia found in clinical grounds is related to the Syndrome of Inappropriate Anti Diuresis (SIAD), which is classified as a hypotonic hyponatremia with a normal EFV. Additional diagnostic criteria of this condition include a urinary osmolality > 100 mOsm/kg and a urinary [Na^+^] > 40 mEq/L with normal dietary salt intake, normal adrenal and thyroid function and no recent use of diuretics [7]. SIAD, which is due in most cases to arginine vasopressin (AVP) release not caused by increased serum osmolality (hence the previous name Syndrome of Inappropriate Anti Diuretic Hormone secretion), may be secondary to a number of different pathologies (e.g. tumors, central nervous system diseases, pulmonary diseases), as well as to treatment with different drugs.

The clinical features of hyponatremia depend on the degree of serum [Na^+^] reduction, but also on the time of onset. In principle, patients with severe acute hyponatremia are more compromised (e.g. seizures, delirium, stupor, coma) than those with chronic hyponatremia (e.g. dizziness, gait alterations, headache, neurocognitive impairment, nausea, vomit). Interestingly, hyponatremia has been associated with bone loss and an increased risk of fractures [8], independent of bone density [9, 10]. Noteworthy, hyponatremia has been identified as an independent risk of death in several morbidities, including cancer [11–14]. Besides its clinical impact, low serum [Na^+^] represents a social and economic burden, because it causes an increased use of healthcare resources and a longer stay in the hospital, overall resulting in higher health-related costs [15].

The treatment of hyponatremia is variable, depending in principle on the EFV and on the severity of symptoms. In severely symptomatic hyponatremia, i.v. hypertonic (3% NaCl) saline solution should be administered, independent of the EFV. In non-critical situations, rehydration with isotonic (0.9% NaCl) saline infusion is the mainstay therapy for hyponatremia with reduced EFV. Hyponatremic patients with normal or increased EFV may be treated with fluid restriction, urea, loop diuretics, vaptans [16, 17]. The use of tolvaptan, the only vaptan available in Europe, has been restricted to patients with hyponatremia secondary to SIAD [18]. More recently, the use of sodium-glucose co-transporter-2 (SGLT-2) inhibitors in patients with SIAD has been advocated [19]. Noteworthy, the rate of serum [Na^+^] correction remains a critical issue, especially in chronic hyponatremia, because of the risk that an increase beyond 8–10 mEq/L in 24 h may cause osmotic demyelination [16, 17, 20, 21].

## COVID-19 and hyponatremia: the numbers

It was in March 2020 that the WHO declared SARS-CoV-2 (COVID-19) infection pandemic. Since then, more than 700 million cases have been confirmed worldwide at the beginning of 2024, with more than 7 million deaths.

Among the hundreds of thousands of manuscripts related to COVID-19 infection, a Medline search using the words “COVID-19” and “hyponatremia” retrieved 229 articles by June 24 2024.

In the 2003 SARS-CoV-1 infection, hyponatremia had been reported in 30–60% of affected patients [22–24]. In COVID-19 infection 20–40% of patients were hyponatremic at hospital admission [25–30]. A meta-analysis that included 23 studies reported a pooled prevalence of hyponatremia among COVID-19 patients of 25.8% [95% CI, 21.8–30.2%] [31].

When hyponatremia was categorized based on the extent of serum [Na^+^] reduction, most frequently mild (serum [Na^+^] 130–134 mEq/L) hyponatremia occurred, as expected. In one study, mild hyponatremia was found in 22% of patients, moderate (serum [Na^+^] 121–129 mEq/L) hyponatremia in 7% of cases, and severe (serum [Na^+^] *≤* 120 mEq/L) in 1% of cases [26]. In another study, 16.7% of patients had a serum [Na^+^] between 130 and 134 mEq/L, 2.5% between 125 and 129 mEq/l, 0.9% between 121 and 124 mEq/L and 0.4% ≤120 mEq/L [25]. In a study authored by the Northwell Nephrology COVID-19 Research Consortium, in 35.5% of patients mild hyponatremia was detected, whereas serum [Na^+^] < 130 mEq/L was reported in 9.1% of cases [28]. In a Ducth study, 25.1% of patients presented with serum [Na^+^] ranging 131–134 mEq/L, 7.5% with serum [Na^+^] ranging 126–130 mEq/L and 1.8% with serum [Na^+^] ≤ 125 mEq/L [30].

One study analyzed retrospective cohort studies, aiming to assess whether hyponatremia in COVID-19 patients persists in the subacute (30–90 days) and chronic (> 90 days) period [32]. Cohorts of patients at high risk of hyponatremia were divided into two groups: COVID-19 patients and no-COVID-19 patients. The authors found that patients with COVID-19 infection had a higher risk to have acute and subacute, but not chronic, hyponatremia than other patients. These findings are in agreement with the idea that, once the inflammation resolves, serum [Na^+^] returns to pre-infection levels.

## Etiopathogenesis of hyponatremia in COVID-19 infection

The identification of the etiopathogenesis of hyponatremia in COVID-19 infection is not obvious. First, as a general consideration, patients may present more than one condition leading to a reduced serum [Na^+^]. In addition, it is well known that a number of drugs can cause hyponatremia. Finally, most of the published studies on COVID-19 and hyponatremia, did not address this issue.

Some reports tried to assess the EFV in hyponatremic COVID-19 patients, based on clinical and biochemical evaluation. Keeping in mind that the heterogeneity of the data is likely due to the fact that the studies have been performed by different groups, the ratio between hyponatremia with reduced or normal EFV in four published studies was 75%/25% [33], 53.9%/46.1% [34], 55.1%/44.9% [35] and 42.8%/57.2% [36].

Overall, **SIAD** has been described as one of the main causes of hyponatremia in COVID-19 infection. Non-osmotically induced AVP secretion appears to be stimulated by pro-inflammatory cytokines released by monocytes and macrophages from infected patients [37–39]. Among these, IL-6 is a known factor that can induce AVP secretion, thus causing SIAD [40, 41]. Accordingly, in IL-6 gene knockout mice, the expression levels of AVP in the paraventricular nucleus were reduced [42]. In the first published study that addressed this issue during the COVID-19 pandemia, we showed the presence of an inverse relationship between serum [Na^+^] and IL-6 in a small cohort of COVID-19 patients [43]. Hyponatremic patients (lowest serum [Na^+^] 128 mEq/L) were divided into two groups: one group received treatment with tocilizumab, a humanized anti-IL-6 receptor antibody, whereas the other group did not. A significant increase in serum [Na^+^] was observed in patients treated with tocilizumab, compared to the control patients. These data further support the role of IL-6 in inducing hyponatremia. It is worth mentioning that tocilizumab has been effectively used in the treatment of seriously ill patients with COVID-19 infection [44]. The inverse relationship between serum [Na^+^] and IL-6 was confirmed in a subsequent study that collected data from 380 patients hospitalized at the Careggi University Hospital in Florence, Italy [29], as well as in a Swiss study that included 184 COVID-19 patients [45]. This study also demonstrated that such a relationship is stronger in COVID-19 infection than in other viral or bacterial infections.

Another possible etiology of SIAD in COVID-19 infection has been related to positive pressure ventilation, which is a known cause of hyponatremia. In this condition SIAD is probably a consequence of non-osmotic stimulation of AVP secretion, which can be induced by pulmonary venous baroreceptors activation in response to a reduction in effective blood volume [46].

Euvolemic hyponatremia in COVID-19 patients has been also associated to **secondary hypocortisolism**. In one study, 40% of 61 patients had low serum ACTH and cortisol levels [47]. The authors suggested that the viral infection might have induced a reversible hypophysitis or a direct hypothalamic damage. Interestingly, angiotensin converting enzyme 2 (ACE2) expression has been detected in different areas of the brain, including the hypothalamus and the pituitary [48]. It is well known that ACE2 has an important role in facilitating the entry of the virus into host cells. It is also worth mentioning that anti-ACTH antibodies have been detected in the serum of COVID-19 patients, which may lead to secondary hypocortisolism. Because similarities in amino acid sequences of SARS-CoV-2 and ACTH have been found, the presence of anti-ACTH antibodies has been suggested as a possible mechanism of the virus to evade the immune response [49].

In addition, **Critical Illness-Related Corticosteroid Insufficiency** (CIRCI), which is defined as a dynamic condition characterized by inappropriate cellular activity of glucocorticoids compared to the severity of the disease, has been associated with COVID-19 infection [50]. A possible explanation for the reduced cortisol secretion was based on the observation that vasopressors were among the treatments used in COVID-19 patients with severe acute respiratory syndrome [51]. ACE2 receptor and transmembrane serine protease 2 immunopositivity was found in adrenocortical cells (zona fasciculata and zona reticularis). Reduced serum cortisol levels were found in the majority of critically ill patients and were possibly attributed to a damage of the zona fasciculata of the adrenal cortex (primary adrenal insufficiency or Addison’s disease) [51]. Other precipitating factors leading to CIRCI in COVID-19 infection have been hypothesized, such as the increased activity of 11-β hydroxysteroid dehydrogenase type 2, which inactivates cortisol, or the decreased expression levels and affinity of cortisol receptors [51].

Another mechanism that can determine serum [Na^+^] reduction is related to fluid loss, due for instance to **vomiting or diarrhea** consequent to viral replication in intestinal epithelial cells [52], with an excessive gastrointestinal sodium loss [53]. A report concluded that about 60% of patients with COVID-19 who presented diarrhea had hyponatremia [54].

**Fig. 2:**
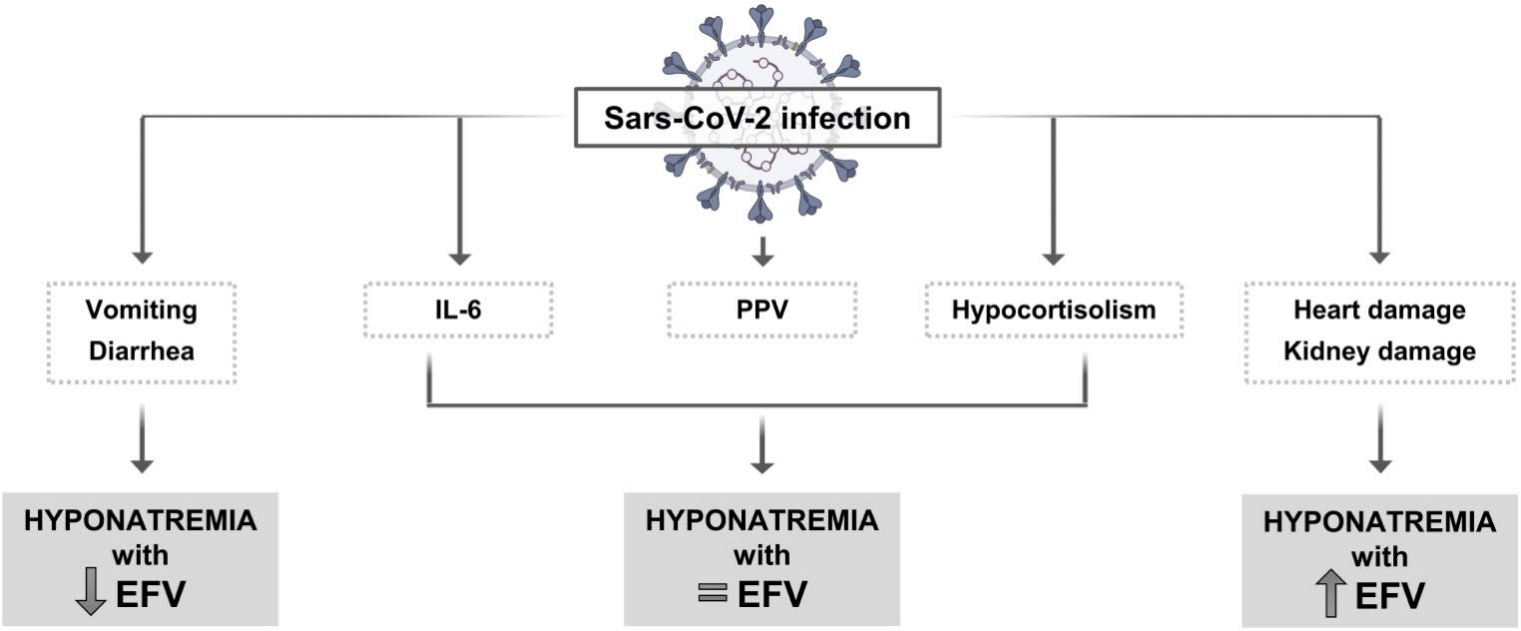
Possible causes of hyponatremia in COVID-19 infection. PPV = positive pressure ventilation; EFV = extracellular fluid volume

Finally, COVID-19-associated **kidney or heart damage**, two conditions known to cause hyponatremia with an increased EFV, may play a role in reducing serum [Na^+^] in affected patients. Viral entry into the kidney in COVID-19 patients is facilitated by the extensive renal localization of ACE2, which the virus employs as a gateway [55]. Viral particles have been detected in the proximal convoluted tubule and in podocytes, thus suggesting a direct renal cell damage [55]. IL-6 release can precipitate acute kidney injury, documented by glomerular alterations, and acute tubular necrosis [55]. In the heart, ACE2 expression in the cell membrane of myocytes facilitates virus access [56]. Cardiomyocyte damage results in muscle hypofunction and heart failure [57] Fig. 2.

## Prognostic value of hyponatremia in COVID-19 patients

Hyponatremia is known to be associated with a worse prognosis in a number of clinical conditions, including cancer, as previously mentioned. Early studies suggested that this was the case also among COVID-19 patients.

In our previously mentioned study, which included a small group of patients, we found a direct relationship between serum [Na^+^] and PaO2/FiO2 ratio at admission and an inverse relationship between serum [Na^+^] and IL-6 levels [43]. Hyponatremia was associated with a more frequent transfer to ICU, need of non-invasive ventilation and death. We suggested that serum [Na^+^] measurement, which is more readily available than IL-6, might be used as a surrogate biomarker of disease severity.

In our subsequent study, which reported data on 380 patients with COVID-19 infection, we confirmed the presence of a direct relationship between serum [Na^+^] and PaO2/FiO2 ratio and an inverse relationship between serum [Na^+^] and IL-6. Again, hyponatremic patients had a more severe disease and a 2.7 fold higher risk of in-hospital mortality than normonatremic ones [29]. In particular, for each mEq/L of serum [Na^+^] reduction, a 14.4% increased risk of death was calculated. Other studies showed that higher IL-6 levels were associated with a greater disease burden [58, 59].

A number of other published manuscripts have addressed the issue of hyponatremia as a predictor of a worse outcome in patients with COVID-19 infection, and in most cases such a relationship was confirmed. One study analyzed many variables possibly associated with a progression to a more severe disease. Hyponatremia, together with pulmonary inflammation indexes, platelet counts, C-reactive protein and prealbumin levels, and PaCO_2_, was among the most powerful variables predicting a heavier disease burden [60]. In another study, a pooled analysis of 1415 COVID-19 patients from 5 different studies, was performed, aiming to assess the relationship between a more severe disease and electrolyte abnormalities. Lower serum [Na^+^], [K^+^] and [Ca^++^] were found to correlate with disease severity [61]. Hyponatremia, hypochloremia and hypocalcemia were associated with a more frequent access to ICU units in a study from Turkey. Here, hyponatremia in particular appeared an independent risk factor for death [62]. Again, in a cohort of Chinese patients, each one of the above mentioned electrolyte disorders was associated with a longer hospitalization [63]. A study performed in New York City, which assessed the relationship between serum [Na^+^] and severity of COVID-19 infection in 4645 patients, found that hyponatremia occurred in 1373 cases [26]. A significant correlation between the severity of hyponatremia and a more negative outcome was found. In particular, patients with moderate (serum [Na^+^] 121–130 mEq/L) and severe (serum [Na^+^] ≤ 120 mEq/L) hyponatremia more frequently required invasive mechanical ventilation and had a longer hospitalization [26].

More recent retrospective studies confirmed the initial findings. A Japanese multicenter cohort study, which reported data from 1055 patients with COVID-19 infection, showed that a lower initial serum [Na^+^] predicted an increased risk of oxygen therapy and of in-hospital death [64]. A meta-analysis of 28 observational studies for a total of 953 COVID-19 patients demonstrated that hyponatremia, hypernatremia or hypocalcemia were associated with a poor COVID-19 outcome, defined as a composite of ICU admission, respiratory aid, acute respiratory distress syndrome and death [65]. The presence of hypocalcemia, as one of the most frequent electrolyte alterations in COVID-19 patients, and its association with a more severe outcome have been reported also by other authors [66, 67]. Interestingly, hypocalcemia and hypovitaminosis D have been related to skeletal complications, including vertebral fractures [67]. It is worth mentioning that hyponatremia has been also addressed as a risk factor for osteoporosis and bone fractures [68] and that the activation of inflammatory immune cells in response to COVID-19 infection has been associated with bone loss [69].

Some studies reported that hyponatremia is more frequent in male COVID-19 patients and that it is associated with a more severe outcome than in females [26, 35, 70].

Interestingly, it was observed that hyponatremia at admission may also affect the health status at follow-up. We performed an observational study in which 6-month follow-up data were collected from 189 patients with a previous hospitalization for COVID-19 infection. Among them, 25% had hyponatremia at admission [71]. In these patients, despite serum [Na^+^] normalization at follow-up, higher levels of LDH, fibrinogen, troponin T and NT-ProBNP were detected compared to those with normonatremia at admission. Furthermore, early hyponatremia was associated with a worse right ventricular function, as assessed by echocardiography, and a greater pulmonary involvement, as indicated by a higher RALE (for Radiographic Assessment of Lung Edema) score.

Another study aimed to determine whether there was an association between hyponatremia correction and patients’ outcome. Among 414 patients admitted for COVID-19 infection, 91 were hyponatremic at admission. Those, in which hyponatremia had been corrected at 72–96 h, had a lower mortality rate compared to those with persistent low serum [Na^+^] [72].

## Management of hyponatremia in COVID-19 patients

The treatment of hyponatremia in COVID-19 patients is in principle based on the known recommendations for the management of hyponatremia [16, 17]. However, it has to be considered that in this setting patients may not be suitable for fluid restriction in view of increased fluid losses due to hyperthermia, vomiting and/or diarrhea, and malnutrition [73]. Although clinical experience on the use of other medications for hyponatremia in COVID-19 patients remains limited, the use of tolvaptan or urea may be considered [73]. To our knowledge, data about the possible use of SGLT-2 inhibitors in these patients are lacking.

In principle, hyponatremia should be virtually considered an acute event in this scenario, unless it is already present for different reasons. Therefore, similarly to hyponatremia caused by etiologies other than COVID-19 infection, patients with acute, symptomatic hyponatremia should receive hypertonic (3% NaCl) saline infusion regardless of the EFV status [16, 17]. According to the published recommendations, in the presence of severe symptoms i.v. boluses (100/150 mL over 10/20 minutes and repeated until an increase in serum [Na^+^] of about 5 mEq/L is obtained) should be used. In such a situation, boluses have been shown to be more effective than i.v. continuous infusion, in terms of a higher increase in serum [Na^+^] and a higher improvement of the Glasgow Coma Scale at 6 and 12 h [74]. However, in COVID-19 patients additional caution should be deserved for severely ill patients, because an excessive rate of infusion might facilitate pulmonary edema [75]. Therefore, continuous i.v. infusion of hypertonic saline solution at a reduced rate might represent a safer approach. Indications on the management of hyponatremia in COVID-19 patients are summarized in Table 1.

**Table 1:** Treatment of hyponatremia in COVID-19 patients

Routine Care	According to guidelines [0.9% NaCl i.v. if EFV is reduced, otherwise fluid restriction (but it may not be possible), urea, loop diuretics, tolvaptan [16, 17, 73]
Acute synthomatic	3% NaCl i.v. ▪ Preferably continuous infusion ▪ Desired limit of serum [Na^+^] correction: 8 mEq/L /24 h or 18 mEq/L /48 h

EFV = extracellular fluid volume

With regard to the 24 h and 48 h limits, published guidelines suggest that serum [Na^+^] increases should be limited to *≤* 8 mEq/L and *≤* 18 mEq/L, respectively [15, 16]. Although the risk of osmotic demyelination syndrome (ODS) is reduced in acute hyponatremia, in patients at high risk of ODS (i.e. serum [Na^+^] *≤* 105 mEq/L, hypokalemia, alcoholism, malnutrition, advanced liver disease), the limit of correction of serum [Na^+^] in the first 24 h should be lowered to 6 mEq/L [16]. A case report-review article published in 2024 retrieved a total of 14 confirmed cases of ODS in patients with COVID-19 infection, in addition to the case presented in the article [76]. However, in most cases neither the initial serum [Na^+^] nor the rate of correction were reported. As a matter of fact, initial hyponatremia was documented only in 4 cases. Among the few cases, in which serum [Na^+^] increase was reported, apparently only in one case the established limits of correction were exceeded (38 mEq/L serum [Na^+^] increase in 48 h). It has to be said, as the authors mention, that the diagnosis of ODS is difficult in ICU, where encephalopathies of different etiologies are rather common. The gold standard for the diagnosis of ODS relies on an MRI assessment. MRI may not be easily feasible in an ICU setting and among the 15 cases of ODS reported in the literature it was performed in only 6 cases. It has to be also added that ODS, which is due to extracellular osmotic changes resulting in oligodendrocyte apoptosis and disruption of myelin sheaths, may be caused by conditions other than rapid serum [Na^+^] correction. These include other electrolyte alterations, such as hypophosphatemia or hypokalemia, hyperglycemia, decreased food intake [77]. Thus, admittedly the association between serum [Na^+^] correction and ODS in COVID-19 patients, as reported in the literature, appears in most cases elusive.

Besides the risk represented by an overly rapid correction of hyponatremia, also inappropriate treatment strategies may lead to negative consequences. This aspect has been covered by an afore mentioned retrospective study, which analyzed 247 patients with COVID-19 infection and hyponatremia (mostly mild), admitted to a tertiary hospital in Madrid, Spain [36]. EFV was clinically assessed in 208 patients and in 57.2% it was considered normal. Most patients with hyponatremia and a normal EFV were incorrectly treated with isotonic saline solution. A higher mortality rate was observed in these patients, compared to those correctly treated or left untreated, which was possibly attributed by the authors to the higher rate of uncorrected hyponatremia, yet not statistically significant, and to fluid overload.

## Conclusions

Hyponatremia is frequently observed in hospitalized patients, and this was also confirmed in patients admitted with COVID-19 infection. In such a scenario, hyponatremia associated with different etiologies was reported at hospital admission in about 25% of patients. Noteworthy, similar to what is known for many other pathological conditions, hyponatremia was associated with a worse outcome also in COVID-19 patients. Hence, this electrolyte alteration deserves careful medical attention also in this condition, in order to use the appropriate treatment strategies, aiming to promptly and safely increase serum [Na^+^].

## Data Availability

No datasets were generated or analysed during the current study.

## References

[1] Hoorn EJ, Lindemans J, Zietse R (2006) Development of severe hyponatraemia in hospitalized patients: treatment-related risk factors and inadequate management. Nephrol Dial Transplant off Publ Eur Dial Transpl Assoc -. Eur Ren Assoc 21:70–76. 10.1093/ndt/gfi082 10.1093/ndt/gfi082 16141458

[2] Ellison DH, Berl T (2007) Clinical practice. The syndrome of inappropriate antidiuresis. N Engl J Med 356:2064–2072. 10.1056/NEJMcp066837 17507705 10.1056/NEJMcp066837

[3] Upadhyay A, Jaber BL, Madias NE (2006) Incidence and prevalence of hyponatremia. Am J Med 119. 10.1016/j.amjmed.2006.05.005. S30-5 10.1016/j.amjmed.2006.05.005 16843082

[4] Adrogué HJ, Madias NE (2000) Hyponatremia. N Engl J Med 342:1581–1589. 10.1056/NEJM200005253422107 10824078 10.1056/NEJM200005253422107

[5] Gennari FJ (1998) Hypo-hypernatraemia: disorders of water balance. Oxf Textb Clin Nephrol 1:175–200

[6] Sterns RH, Narins RG (1991) Hypernatremia and hyponatremia: pathophysiology, diagnosis, and therapy. Contemp Manag Crit care 1:161–191

[7] Schwartz WB, Bennett W, Bartter Curelops FC (1957) A syndrome of renal sodium loss and hyponatremia probably resulting from inappropriate secretion of antidiuretic hormone. Am J Med 23:529–542. 10.1016/0002-9343(57)90224-3 13469824 10.1016/0002-9343(57)90224-3

[8] Kinsella S, Moran S, Sullivan MO et al (2010) Hyponatremia independent of osteoporosis is associated with fracture occurrence. Clin J Am Soc Nephrol 5:275–280. 10.2215/CJN.06120809 20056759 10.2215/CJN.06120809 PMC2827601

[9] Verbalis JG, Barsony J, Sugimura Y et al (2010) Hyponatremia-induced osteoporosis. J bone Min Res off J Am Soc Bone Min Res 25:554–563. 10.1359/jbmr.090827 10.1359/jbmr.090827 PMC3153395 19751154

[10] Barsony J, Sugimura Y, Verbalis JG (2011) Osteoclast response to low extracellular sodium and the mechanism of hyponatremia-induced bone loss. J Biol Chem 286:10864–10875. 10.1074/jbc.M110.155002 21135109 10.1074/jbc.M110.155002 PMC3060537

[11] Wald R, Jaber BL, Price LL et al (2010) Impact of hospital-associated hyponatremia on selected outcomes. Arch Intern Med 170:294–302. 10.1001/archinternmed.2009.513 20142578 10.1001/archinternmed.2009.513

[12] Corona G, Giuliani C, Parenti G et al (2013) Moderate hyponatremia is associated with increased risk of mortality: evidence from a meta-analysis. PLoS ONE 8:e80451. 10.1371/journal.pone.0080451 24367479 10.1371/journal.pone.0080451 PMC3867320

[13] Mohan S, Gu S, Parikh A, Radhakrishnan J (2013) Prevalence of hyponatremia and association with mortality: results from NHANES. Am J Med 126:1127–37e1. 10.1016/j.amjmed.2013.07.021 24262726 10.1016/j.amjmed.2013.07.021 PMC3933395

[14] Holland-Bill L, Christiansen CF, Heide-Jørgensen U et al (2015) Hyponatremia and mortality risk: a Danish cohort study of 279 508 acutely hospitalized patients. Eur J Endocrinol 173:71–81. 10.1530/EJE-15-0111 26036812 10.1530/EJE-15-0111

[15] Corona G, Giuliani C, Parenti G et al (2016) The Economic Burden of Hyponatremia: systematic review and Meta-analysis. Am J Med 129:823–835e4. 10.1016/j.amjmed.2016.03.007 27059386 10.1016/j.amjmed.2016.03.007

[16] Verbalis JG, Goldsmith SR, Greenberg A et al (2013) Diagnosis, evaluation, and treatment of hyponatremia: expert panel recommendations. Am J Med 126:S1–42. 10.1016/j.amjmed.2013.07.006 24074529 10.1016/j.amjmed.2013.07.006

[17] Spasovski G, Vanholder R, Allolio B et al (2014) Clinical practice guideline on diagnosis and treatment of hyponatraemia. Eur J Endocrinol 170:G1–47. 10.1530/EJE-13-1020 24569125 10.1530/EJE-13-1020

[18] Peri A (2013) Clinical review: the use of vaptans in clinical endocrinology. J Clin Endocrinol Metab 98:1321–1332. 10.1210/jc.2012-4082 23401044 10.1210/jc.2012-4082

[19] Sarafidis P, Loutradis C, Ferro CJ, Ortiz A (2020) SGLT-2 inhibitors to Treat Hyponatremia Associated with SIADH: a novel indication? Am J Nephrol 51:553–555 32645703 10.1159/000509082

[20] Corona G, Simonetti L, Giuliani C et al (2014) A case of osmotic demyelination syndrome occurred after the correction of severe hyponatraemia in hyperemesis gravidarum. BMC Endocr Disord 14:34. 10.1186/1472-6823-14-34 24725751 10.1186/1472-6823-14-34 PMC3989779

[21] Adrogué HJ, Tucker BM, Madias NE (2022) Diagnosis and management of Hyponatremia: a review. JAMA 328:280–291. 10.1001/jama.2022.11176 35852524 10.1001/jama.2022.11176

[22] Leong H-N, Earnest A, Lim H-H et al (2006) SARS in Singapore–predictors of disease severity. Ann Acad Med Singap 35:326–331 16829999

[23] Leong H-N, Chan K-P, Oon LLE et al (2006) Clinical and laboratory findings of SARS in Singapore. Ann Acad Med Singap 35:332–339 16830000

[24] Choi JS, Bae EH, Ma SK et al (2015) Prognostic impact of hyponatraemia in patients with colorectal cancer. Color Dis off J Assoc Coloproctology Gt Br Irel 17:409–416. 10.1111/codi.12878 10.1111/codi.12878 25524356

[25] Ruiz-Sánchez JG, Núñez-Gil IJ, Cuesta M et al (2020) Prognostic impact of Hyponatremia and Hypernatremia in COVID-19 Pneumonia. A HOPE-COVID-19 (Health Outcome Predictive evaluation for COVID-19) Registry Analysis. Front Endocrinol (Lausanne) 11:599255. 10.3389/fendo.2020.599255 33329400 10.3389/fendo.2020.599255 PMC7734292

[26] Frontera JA, Valdes E, Huang J et al (2020) Prevalence and impact of hyponatremia in patients with Coronavirus Disease 2019 in New York City. Crit Care Med 48:e1211–e1217. 10.1097/CCM.0000000000004605 32826430 10.1097/CCM.0000000000004605 PMC7467047

[27] Atila C, Sailer CO, Bassetti S et al (2021) Prevalence and outcome of dysnatremia in patients with COVID-19 compared to controls. Eur J Endocrinol 184:409–418. 10.1530/EJE-20-1374 33449918 10.1530/EJE-20-1374 PMC9494345

[28] Hirsch JS, Uppal NN, Sharma P et al (2021) Prevalence and outcomes of hyponatremia and hypernatremia in patients hospitalized with COVID-19. Nephrol Dial Transplant off Publ Eur Dial Transpl Assoc -. Eur Ren Assoc 36:1135–1138. 10.1093/ndt/gfab067 10.1093/ndt/gfab067 PMC7989196 33724428

[29] Berni A, Malandrino D, Corona G et al (2021) Serum sodium alterations in SARS CoV-2 (COVID-19) infection: impact on patient outcome. Eur J Endocrinol 185:137–144. 10.1530/EJE-20-1447 33950864 10.1530/EJE-20-1447 PMC9494309

[30] de Haan L, Ten Wolde M, Beudel M et al (2023) What is the aetiology of dysnatraemia in COVID-19 and how is this related to outcomes in patients admitted during earlier and later COVID-19 waves? A multicentre, retrospective observational study in 11 Dutch hospitals. BMJ Open 13:e075232. 10.1136/bmjopen-2023-075232 37963704 10.1136/bmjopen-2023-075232 PMC10649520

[31] Kadir F, Yeap BT, Hayati F et al (2022) Medical education during the COVID-19: a Malaysian experience. Int J Med Educ 13:84–87. 10.5116/ijme.6231.a20e 35366644 10.5116/ijme.6231.a20e PMC9017504

[32] Achinger SG, Kumar A, Tsalatsanis A (2024) COVID-19 and incidence of Acute and Chronic Hyponatremia: a matched cohort study. Kidney Med 6:100750. 10.1016/j.xkme.2023.100750 38259724 10.1016/j.xkme.2023.100750 PMC10801209

[33] Tzoulis P, Waung JA, Bagkeris E et al (2021) Dysnatremia is a predictor for morbidity and mortality in hospitalized patients with COVID-19. J Clin Endocrinol Metab 106:1637–1648. 10.1210/clinem/dgab107 33624101 10.1210/clinem/dgab107 PMC7928894

[34] Ruiz-Sánchez JG, Chávez-Díaz P, Cárdenas-Salas J (2022) The evolution of Hypovolemic and Euvolemic Hyponatremia coincides with an inflammatory status in patients with COVID-19: an Observational Cohort Study. Endocrines 3:726–735

[35] Chan GCK, Wong CK, So BYF et al (2022) Epidemiology and outcomes of hyponatremia in patients with COVID-19-A territory-wide study in Hong Kong. Front Med 9:1096165. 10.3389/fmed.2022.1096165 10.3389/fmed.2022.1096165 PMC9874105 36714113

[36] Pazos-Guerra M, Ruiz-Sánchez JG, Pérez-Candel X et al (2023) Inappropriate therapy of euvolemic hyponatremia, the most frequent type of hyponatremia in SARS-CoV-2 infection, is associated with increased mortality in COVID-19 patients. Front Endocrinol (Lausanne) 14:1227059. 10.3389/fendo.2023.1227059 37560297 10.3389/fendo.2023.1227059 PMC10408442

[37] Liu J, Li S, Liu J et al (2020) Longitudinal characteristics of lymphocyte responses and cytokine profiles in the peripheral blood of SARS-CoV-2 infected patients. EBioMedicine 55:102763. 10.1016/j.ebiom.2020.102763 32361250 10.1016/j.ebiom.2020.102763 PMC7165294

[38] Hu B, Huang S, Yin L (2021) The cytokine storm and COVID-19. J Med Virol 93:250–256. 10.1002/jmv.26232 32592501 10.1002/jmv.26232 PMC7361342

[39] McGonagle D, Sharif K, O’Regan A, Bridgewood C (2020) The role of cytokines including Interleukin-6 in COVID-19 induced Pneumonia and Macrophage Activation Syndrome-Like Disease. Autoimmun Rev 19:102537. 10.1016/j.autrev.2020.102537 32251717 10.1016/j.autrev.2020.102537 PMC7195002

[40] Mastorakos G, Weber JS, Magiakou MA et al (1994) Hypothalamic-pituitary-adrenal axis activation and stimulation of systemic vasopressin secretion by recombinant interleukin-6 in humans: potential implications for the syndrome of inappropriate vasopressin secretion. J Clin Endocrinol Metab 79:934–939. 10.1210/jcem.79.4.7962300 7962300 10.1210/jcem.79.4.7962300

[41] Swart RM, Hoorn EJ, Betjes MG, Zietse R (2011) Hyponatremia and inflammation: the emerging role of interleukin-6 in osmoregulation. Nephron Physiol 118:45–51. 10.1159/000322238 21196778 10.1159/000322238

[42] Benrick A, Schéle E, Pinnock SB et al (2009) Interleukin-6 gene knockout influences energy balance regulating peptides in the hypothalamic paraventricular and supraoptic nuclei. J Neuroendocrinol 21:620–628. 10.1111/j.1365-2826.2009.01879.x 19490366 10.1111/j.1365-2826.2009.01879.x

[43] Berni A, Malandrino D, Parenti G et al (2020) Hyponatremia, IL-6, and SARS-CoV-2 (COVID-19) infection: may all fit together? J Endocrinol Invest 43:1137–1139. 10.1007/s40618-020-01301-w 32451971 10.1007/s40618-020-01301-w PMC7246958

[44] Luo P, Liu Y, Qiu L et al (2020) Tocilizumab treatment in COVID-19: a single center experience. J Med Virol 92:814–818. 10.1002/jmv.25801 32253759 10.1002/jmv.25801 PMC7262125

[45] Atila C, Monnerat S, Bingisser R et al (2022) Inverse relationship between IL-6 and sodium levels in patients with COVID-19 and other respiratory tract infections: data from the COVIVA study. Endocr Connect 11: 10.1530/EC-22-0171 10.1530/EC-22-0171 PMC9578076 36006851

[46] Jones DP (2018) Syndrome of Inappropriate Secretion of antidiuretic hormone and hyponatremia. Pediatr Rev 39:27–35. 10.1542/pir.2016-0165 29292284 10.1542/pir.2016-0165

[47] Leow MK-S, Kwek DS-K, Ng AW-K et al (2005) Hypocortisolism in survivors of severe acute respiratory syndrome (SARS). Clin Endocrinol (Oxf) 63:197–202. 10.1111/j.1365-2265.2005.02325.x 16060914 10.1111/j.1365-2265.2005.02325.x PMC7188349

[48] Frara S, Loli P, Allora A et al (2022) COVID-19 and hypopituitarism. Rev Endocr Metab Disord 23:215–231. 10.1007/s11154-021-09672-y 34387832 10.1007/s11154-021-09672-y PMC8363093

[49] Pérez-Torres D, Díaz-Rodríguez C, Armentia-Medina A (2022) Anti-ACTH antibodies in critically ill Covid-19 patients: a potential immune evasion mechanism of SARS-CoV-2. Med Intensiva 46:472–474 34642524 10.1016/j.medin.2021.09.002 PMC8498650

[50] Marino LO, Souza HP (2020) Dysfunction of the hypothalamic-pituitary-adrenal axis in critical illness: a narrative review for emergency physicians. Eur J Emerg Med off J Eur Soc Emerg Med 27:406–413. 10.1097/MEJ.0000000000000693 10.1097/MEJ.0000000000000693 PMC7587239 33108130

[51] Mao Y, Xu B, Guan W et al (2020) The adrenal cortex, an underestimated site of SARS-CoV-2 infection. Front Endocrinol (Lausanne) 11:593179. 10.3389/fendo.2020.593179 33488517 10.3389/fendo.2020.593179 PMC7820749

[52] Choi KW, Chau TN, Tsang O et al (2003) Outcomes and prognostic factors in 267 patients with severe acute respiratory syndrome in Hong Kong. Ann Intern Med 139:715–723. 10.7326/0003-4819-139-9-200311040-00005 14597455 10.7326/0003-4819-139-9-200311040-00005

[53] Habas E, Ali E, Habas A et al (2022) Hyponatremia and SARS-CoV-2 infection: a narrative review. Med (Baltim) 101:e30061. 10.1097/MD.0000000000030061 10.1097/MD.0000000000030061 PMC9370252 35960124

[54] Ata F, Almasri H, Sajid J, Yousaf Z (2020) COVID-19 presenting with diarrhoea and hyponatraemia. BMJ Case Rep 13. 10.1136/bcr-2020-235456 10.1136/bcr-2020-235456 PMC10577739 32513768

[55] Batlle D, Soler MJ, Sparks MA et al (2020) Acute kidney Injury in COVID-19: emerging evidence of a distinct pathophysiology. J Am Soc Nephrol 31:1380–1383. 10.1681/ASN.2020040419 32366514 10.1681/ASN.2020040419 PMC7350999

[56] Hu W, Lv X, Li C et al (2021) Disorders of sodium balance and its clinical implications in COVID-19 patients: a multicenter retrospective study. Intern Emerg Med 16:853–862. 10.1007/s11739-020-02515-9 33064253 10.1007/s11739-020-02515-9 PMC7563904

[57] Bader F, Manla Y, Atallah B, Starling RC (2021) Heart failure and COVID-19. Heart Fail Rev 26:1–10. 10.1007/s10741-020-10008-2 32720082 10.1007/s10741-020-10008-2 PMC7383122

[58] Diao B, Wang C, Tan Y et al (2020) Reduction and functional exhaustion of T cells in patients with Coronavirus Disease 2019 (COVID-19). Front Immunol 11:827. 10.3389/fimmu.2020.00827 32425950 10.3389/fimmu.2020.00827 PMC7205903

[59] Han H, Ma Q, Li C et al (2020) Profiling serum cytokines in COVID-19 patients reveals IL-6 and IL-10 are disease severity predictors. Emerg Microbes Infect 9:1123–1130. 10.1080/22221751.2020.1770129 32475230 10.1080/22221751.2020.1770129 PMC7473317

[60] Duan J, Wang X, Chi J et al (2020) Correlation between the variables collected at admission and progression to severe cases during hospitalization among patients with COVID-19 in Chongqing. J Med Virol 92:2616–2622. 10.1002/jmv.26082 32470186 10.1002/jmv.26082 PMC7283752

[61] Lippi G, South AM, Henry BM (2020) Electrolyte imbalances in patients with severe coronavirus disease 2019 (COVID-19). Ann Clin Biochem 57:262–265. 10.1177/0004563220922255 32266828 10.1177/0004563220922255 PMC8173320

[62] Tezcan ME, Dogan Gokce G, Sen N et al (2020) Baseline electrolyte abnormalities would be related to poor prognosis in hospitalized coronavirus disease 2019 patients. New Microbes new Infect 37:100753. 10.1016/j.nmni.2020.100753 32904987 10.1016/j.nmni.2020.100753 PMC7462442

[63] Wu Y, Hou B, Liu J et al (2020) Risk factors Associated with Long-Term hospitalization in patients with COVID-19: a Single-Centered, Retrospective Study. Front Med 7:315. 10.3389/fmed.2020.00315 10.3389/fmed.2020.00315 PMC7296106 32582749

[64] Hata T, Goto T, Yamanaka S et al (2024) Prognostic value of initial serum sodium level in predicting disease severity in patients with COVID-19: a multicenter retrospective study. J Infect Chemother off J Japan Soc Chemother 30:181–187. 10.1016/j.jiac.2023.10.002 10.1016/j.jiac.2023.10.002 37802152

[65] Song HJJMD, Chia AZQ, Tan BKJ et al (2023) Electrolyte imbalances as poor prognostic markers in COVID-19: a systemic review and meta-analysis. J Endocrinol Invest 46:235–259. 10.1007/s40618-022-01877-5 36070177 10.1007/s40618-022-01877-5 PMC9449297

[66] Di Filippo L, Formenti AM, Rovere-Querini P et al (2020) Hypocalcemia is highly prevalent and predicts hospitalization in patients with COVID-19. Endocrine 68:475–478 32533508 10.1007/s12020-020-02383-5 PMC7292572

[67] di Filippo L, Doga M, Frara S, Giustina A (2022) Hypocalcemia in COVID-19: prevalence, clinical significance and therapeutic implications. Rev Endocr Metab Disord 23:299–308. 10.1007/s11154-021-09655-z 33846867 10.1007/s11154-021-09655-z PMC8041474

[68] Murthy K, Ondrey GJ, Malkani N, The effects of hyponatremia on bone density and fractures: a systematic review and meta-analysis (2019) Endocr Pract off J Am Coll Endocrinol Am Assoc Clin Endocrinol 25:366–378. 10.4158/EP-2018-0499 10.4158/EP-2018-0499 30720342

[69] Sapra L, Saini C, Garg B et al (2022) Long-term implications of COVID-19 on bone health: pathophysiology and therapeutics. Inflamm Res off J Eur Histamine Res Soc [et al] 71:1025–1040. 10.1007/s00011-022-01616-9 10.1007/s00011-022-01616-9 PMC9330992 35900380

[70] Khidir RJY, Ibrahim BAY, Adam MHM et al (2022) Prevalence and outcomes of hyponatremia among COVID-19 patients: a systematic review and meta-analysis. Int J Health Sci (Qassim) 16:69–84 36101848 PMC9441642

[71] Malandrino D, Berni A, Fibbi B et al (2023) Relationship between hyponatremia at hospital admission and cardiopulmonary profile at follow-up in patients with SARS-CoV-2 (COVID-19) infection. J Endocrinol Invest 46:577–586. 10.1007/s40618-022-01938-9 36284058 10.1007/s40618-022-01938-9 PMC9595583

[72] de La Flor JC, Gomez-Berrocal A, Marschall A et al (2022) The impact of the correction of hyponatremia during hospital admission on the prognosis of SARS-CoV-2 infection. Med Clin (English ed) 159:12–18. 10.1016/j.medcle.2021.07.021 10.1016/j.medcle.2021.07.021 PMC9240945 35784827

[73] Tzoulis P, Grossman AB, Baldeweg SE et al (2021) Management of endocrine disease: Dysnatraemia in COVID-19: prevalence, prognostic impact, pathophysiology, and management. Eur J Endocrinol 185:R103–R111. 10.1530/EJE-21-0281 34370712 10.1530/EJE-21-0281 PMC8428074

[74] Garrahy A, Dineen R, Hannon AM et al (2019) Continuous Versus Bolus infusion of Hypertonic saline in the treatment of symptomatic hyponatremia caused by SIAD. J Clin Endocrinol Metab 104:3595–3602. 10.1210/jc.2019-00044 30882872 10.1210/jc.2019-00044

[75] Christ-Crain M, Hoorn EJ, Sherlock M et al (2021) Endocrinology in the time of COVID-19-2021 updates: the management of diabetes insipidus and hyponatraemia. Eur J Endocrinol 185:G35–G42. 10.1530/EJE-21-0596 34292875 10.1530/EJE-21-0596 PMC8428073

[76] Dimitrov G, Naeva D, Duparinova R et al (2024) Osmotic demyelination syndrome amidst COVID-19: a case report with literature review. Neurologia 39:213–217 38460994 10.1016/j.nrleng.2023.11.001

[77] Corona G, Giuliani C, Verbalis JG et al (2015) Hyponatremia improvement is associated with a reduced risk of mortality: evidence from a meta-analysis. PLoS One 10:e0124105. 10.1371/journal.pone.0124105 25905459 10.1371/journal.pone.0124105 PMC4408113

